# A cross-sectional study of factors influencing sexual health among spouses of patients with cervical cancer

**DOI:** 10.1371/journal.pone.0322141

**Published:** 2025-05-29

**Authors:** Yan Shi, Li Zhang, Yu Zhou, Xuejun Liao, Tingting Zhu, Jun Cai

**Affiliations:** Department of Gynecology, The Second Affiliated Hospital of Chongqing Medical University, Chongqing, China; Iranian Institute for Health Sciences Research, IRAN, ISLAMIC REPUBLIC OF

## Abstract

**Background:**

After treatment, cervical cancer patients commonly experience sexual health problems that lead to marital conflict. However, the sexual health cognition, distress and needs of patients’ spouses remain unclear. This study examined the factors influencing sexual health among spouses of patients with cervical cancer and provided a reference for targeted nursing interventions.

**Methods:**

This cross-sectional study was conducted in Chongqing, China. A total of 202 spouses of patients with cervical cancer were selected via convenience sampling. All participants completed the questionnaire via WeChat from September 27 to October 5, 2022. The questionnaire assessed demographic information, clinical information, and information about sexual health cognition, sexual distress and sexual needs. Multiple linear regression analysis was used to identify the factors associated with sexual health cognition. Pearson’s correlation analysis was used to analyse the correlations among sexual cognition, distress and needs.

**Results:**

The cognitive scores of the spouses of cervical cancer patients with respect to sexual health ranged from 6 to 25 (13.79 ± 6.74). The distress scores ranged from 8 to 35 (27.24 ± 7.88). The need scores ranged from 3 to 12 (8.68 ± 3.64). Age, education level, place of residence and other factors affected sexual health cognition. Age (β, -0.178; 95% CI, 0.099–1.060), education level (β, 0.152; 95% CI, 0.021–0.663), place of residence (β, 0.665; 95% CI, 0.102–5.789), occupation type (β, 0.507; 95% CI, 0.485–4.982), and monthly family income (β, 0.229; 95% CI, 0.311–1.344) were associated with higher levels of sexual health cognition. The results of Pearson’s correlation analysis revealed that there was a negative correlation between sexual health cognition and distress (r = - 0.6165, 95% CI: -0.69 to -0.52, P < 0.001), that cognition was positively correlated with needs (r = 0.6757,95% CI 95% CI: 0.59 to 0.74, P < 0.001), and that distress was positively correlated with needs (r = 0.6860, 95% CI: 0.60 to 0.75, P < 0.001).

**Conclusions:**

Our study revealed that sexual health cognition among spouses of patients with cervical cancer is affected by multiple factors. The degree of sexual distress among these individuals is high. There was a negative correlation between sexual cognition and distress, a positive correlation between sexual cognition and demand, and a positive correlation between sexual distress and demand. These results suggest that nurses should also include the patient’s spouse in the care process and provide targeted sexual health guidance, thereby improving the overall quality of life of patients and spouses.

## Introduction

Cervical cancer is the fourth most common cancer in women, and approximately 600 000 cases of cervical cancer were reported in 2020 [1]. China is currently one of the countries with the highest incidence of cervical cancer, with approximately 65,000 new cases of cervical cancer and 25,000 cervical cancer-related deaths every year [2]. The incidence of cervical cancer in Chongqing increased from 8.03 out of 100,000 in 2010 to 21.60 out of 100,000 in 2021, and the mortality rate increased from 3.77 out of 100,000 to 5.00 out of 100,000. The incidence, mortality and disease burden are higher than the national average and show an increasing trend annually, especially in rural areas, where there is a trend towards a younger age at onset [3,4].

With the advancements in effective screening and treatment methods, the prognosis of patients with cervical cancer has improved significantly, and the overall 5-year survival rate is 70% [5]. However, cervical cancer and its treatment can impair a patient’s sexual function and this deficit may persist for a long time [6,7]. Therefore, improving the sexual health of survivors is considered an important component of improving their quality of life [7]. However, during clinical treatment for cervical cancer, medical staff and patients are unlikely to consider the complication of sexual dysfunction [8,9].

As early as 2002, the WHO defined sexual health as follows: sexual health must be seen as a state of physical, emotional, mental and social well-being related to sex, not merely the absence of disease, dysfunction or infirmity [10]. The WHO indicated that sexual life constitutes a multivariate equation of physiological and psychosocial determinants that must be evaluated clinically. Sex is an important aspect of existence that promotes happiness, self-esteem and general resilience; coping, persistence, and survival skills can be strengthened for people with chronic diseases; thus, health care professionals (HCPs) should routinely address sexual issues [11].

Sexual function in cervical cancer survivors declines significantly after treatment, regardless of the therapeutic approach used. Only a few studies have examined psychosexual needs, perceptions, and the acceptance of support among cervical cancer survivors [12]. Sexual behaviour and intimate relationships are important issues for survivors [13]. As the primary caregiver and the most important source of support for women diagnosed with gynaecological cancer, spouses can help women meet their medical and nonmedical needs and manage their conditions [14]. Teskereci et al. reported that a substantial majority of caregivers of individuals with gynaecological cancers were identified as their partners [15]. It has been suggested that sexual health and having a close relationship with a partner are valuable “safe havens” that provide emotional support during the cancer experience. However, this psychological buffer can be disrupted as the disease progresses [15,16]. Cancer affects not only patients’ physical health but also their sexual behaviour and intimate relationships with their partners [17]. There is still a lack of attention devoted to the sexual health of the spouses of patients with cervical cancer.

At present, most related research has focused on the sexual health of cervical cancer patients [18–20]. Our previous research revealed that cervical cancer patients in Chongqing lack sexual health knowledge and experience disharmonious relationships with their spouses, which has an effect on their family relationships. There is considerable demand for sexual health knowledge among patients, but medical staff have not devoted sufficient attention to this demand, and there is a lack of systematic management plans [21]. The breakdown of relationships is mostly related to a lack of sexual compatibility; however, cancer services have traditionally focused on patient care [22], whereas the recognition of research about spouses’ sexual health is usually explored to a lesser degree [23,24]. Furthermore, previous studies have shown that it is important to focus on providing support to people who have been diagnosed with cancer and their spouses to enhance the benefit of sharing their mutual posttraumatic growth [25,26]. Therefore, health care workers should pay attention to the sexual health of patients while not ignoring the sexual health of patients’ spouses.

The objective of this study was to assess the sexual health cognition, sexual distress and sexual health-related knowledge needs of the spouses of cervical cancer patients through a questionnaire survey. The influencing factors associated with the sexual health cognition of patients’ spouses and the need for sexual health-related knowledge were determined to provide a basis for establishing sexual health intervention programs for these professionals. In this study, we hypothesized that sexual health-related knowledge needs are associated with sexual health cognition and sexual distress.

## Methods

### Design and sample

This cross-sectional study was conducted in three tertiary hospitals in Chongqing, China. The spouses of patients with cervical cancer were recruited via convenience sampling. The inclusion criteria were as follows: were married, aged between 18 and 55 years, were spouses of patients who were diagnosed with cervical cancer, the spouse was treated at least 3 months prior, and the participant and patient had resumed having sex. The exclusion criteria were as follows: a) had chronic diseases such as diabetes, heart disease, or hypertension; b) were unable to understand or complete the questionnaires; or c) had a history of sexual dysfunction.

The data were collected between September 27 and October 5, 2022. This study was reported in accordance with the Consolidated Standards of Reporting Trials (STROBE) statement, which was adopted from Skrivankova, Richmond, and Woolf (2021) [27]. The investigators underwent uniform training. The questionnaires were administered via WeChat, and the investigators provided explanations and clarifications as necessary.

The professional questionnaire survey platform “Wenjuan Xing” was used. The respondents logged in via WeChat and completed the survey anonymously. The survey took approximately 10–20 minutes to complete. The network automatically screened out invalid questionnaires, including duplicate surveys completed from the same account and questionnaires that were completed within 5 minutes. In this study, a homemade questionnaire was administered with the approval of the research ethics committees of the participating hospitals. The participants were informed about the aims of the study and the importance of enrolment. A total of 208 spouses answered the questionnaire. After excluding 6 unqualified participants who did not complete the questionnaire or did not complete it correctly, the valid questionnaire rate was 89.77% (202).

This study was approved by the Ethics Committee of the Second Affiliated Hospital of Medical University in Chongqing, China (No. 2021.304). It was registered with the Chinese Clinical Trial Registry with the number ChiCTR2200056456.

The first section of the questionnaire mainly included informed consent. The participants read the informed consent form, and if they agreed to participate in the study, they clicked “I agree” to advance to the survey. The data were collected anonymously, and personal information was not disclosed, except demographic data. All the raw data were kept by the researcher.

### Measures

#### Demographic characteristics.

Demographic data, including age, education level, place of residence, occupation type, family monthly income, disease type, treatment methods, years of living with spouse, and marital status, were collected.

#### Spouses’ perceptions of sexual problems, distress and needs questionnaire.

The questionnaire was designed by referring to the relevant literature [28]. It included 18 items assessing the three dimensions of the cognition, distress and needs of spouses with respect to sexual problems: a) Six items assess spouses’ understanding of sexual health issues, including whether they can engage in sexual behaviour after the cancer diagnosis and treatment, contraindications, appropriate times, sexual lifestyle changes, common problems and treatment measures. A 5-point Likert scale was adopted, ranging from “strongly disagree (1 point)” to “strongly agree (5 points)”, and the total score ranged from 6–30 points. The higher the score was, the greater the spouse’s awareness of sexual issues was. b) Eight items were used to assess the degree of distress among spouses with respect to sexual health problems. A 5-point Likert scale was used, ranging from “strongly disagree (1 point)” to “strongly agree (5 points)”. The total scores ranged from 8–40 points. The higher the score was, the greater the degree of sexual distress with respect to sexual health problems. c) Four items were used to assess spouses’ need for sexual health-related knowledge. The first 3 items were scored on a 5-point Likert scale ranging from “strongly disagree (1 point)” to “strongly agree (5 points)”, and the total possible score ranged from 3–15 points. The last item was a multiple-choice question that assessed the guidance models needed by the spouse, with a higher percentage of choices indicating greater need.

#### Questionnaire reliability and validity measurement.

Eight experts with a bachelor’s degree or above and associate senior or above titles in related fields were selected to evaluate the questionnaire items. After 2 rounds of modification and evaluation, the questionnaire was finalized. The content validity index was 0.851. Twelve respondents were randomly selected. In the preliminary survey, the Cronbach’s α coefficient of the questionnaire was 0.873, the Cronbach’s α coefficient of each dimension was 0.826 ~ 0.859, and the retest reliability was 0.838. Exploratory factor analysis revealed that the factor loadings ranged from 0.631 to 0.774, and the cumulative variance contribution rate was 64.583%, which was higher than the 40% threshold [29], thus indicating the good reliability and validity of the questionnaire.

### Statistical analyses

The data were analysed via SPSS software version 22.0. Descriptive statistics, including age, education level, place of residence, and occupation type, were used to determine the distribution of demographic characteristics. Continuous variables are reported as means and standard deviations. Categorical variables are expressed as absolute values and percentages. Multiple linear regression analysis was used to measure the influencing factors associated with sexual health cognition. Pearson’s correlation analysis was used to analyse the correlations among sexual cognition, distress and need.

## Results

### Demographic characteristics

A total of 202 spouses were enrolled, and their demographic characteristics are summarized in Table 1. Most of the participants (82.2%) were aged between 30 and 50 years, and 34.7% of the participants had a college degree or above. A total of 71.8% of the participants were office workers, and more than 50% lived in rural areas.

**Table 1:** Demographic characteristics of the study subjects (n = 202).

Characteristic		n (%)
Age, yr	＜30	2 (1.0)
	30-40	48 (23.8)
	41-50	118 (58.4)
	＞50	34 (16.8)
Education level	Primary school or below	37 (18.3)
	Secondary/high school	95 (47.0)
	Associate degree	42 (20.8)
	Bachelor’s degree or above	28 (13.9)
Place of residence	Urban	83 (41.1)
	Rural	119 (58.9)
Occupation type	Office worker^a^	145 (71.8)
	Manual worker^b^	57 (28.2)
Monthly family income, RMB	＜3000	34 (16.8)
	3000-8000	133 (65.8)
	＞8000	35 (17.3)
FIGO^c^ stage of your spouse	IA1 ~ IB3	78 (38.6)
	IIA1 ~ IIB	104 (51.5)
	IIIA~IIIC	20 (9.9)
Disease type of your spouse	Squamous cell carcinoma	181 (89.6)
	Glandular cancer	18 (8.9)
	Other	3 (1.5)
Treatment methods of your spouse	Surgery	15 (7.4)
	Operation +chemotherapy	160 (79.2)
	Operation +radiotherapy	8 (4.0)
	Operation +chemoradiotherapy	19 (9.4)
Marital status	Primary marriage	194 (96.0)
	Digamy	8 (4.0)
How long have you been married？yrs	<3	3 (1.5)
	3 ~ 5	24 (11.9)
	>5	175 (86.6)

^a^Refers to workers engaged in sedentary thinking and intellectual activities.

^b^Refers to workers who rely on physical strength for productive labour.

^c^International Federation of Gynecology and Obstetrics

### The current situation of sexual health cognition

The cognitive scores of the spouses with respect to sexual health ranged from 6 to 25 (13.79 ± 6.74). These data are summarized in Table 2.

**Table 2:** The cognitive scores of the spouses of patients with cervical cancer with respect to sexual health (n = 202).

Variables	Strongly Disagree, n (%)	Disagree, n (%)	Neither agree nor disagree, n (%)	Agree, n (%)	Strongly Agree, n (%)
You can have sex after a cancer diagnosis and treatment	40 (19.8)	45 (22.3)	26 (12.8)	44 (21.8)	47 (23.3)
You know the contraindications to a sexual life after cancer diagnosis and treatment	42 (20.8)	44 (21.8)	30 (14.9)	45 (22.3)	41 (20.2)
You know the right time to have sex after a cancer diagnosis and treatment	39 (19.3)	47 (23.3)	33 (16.3)	44 (21.8)	39 (19.3)
You know about sexual lifestyle changes that occur after a cancer diagnosis and treatment	35 (17.3)	38 (18.8)	23 (11.4)	51 (25.3)	55 (27.2)
You know you will have problems in your sex life after a cancer diagnosis and treatment	32 (15.8)	36 (17.9)	16 (7.9)	55 (27.2)	63 (31.2)
You know how to deal with common problems of sex after cancer diagnosis and treatment	49 (24.3)	52 (25.7)	29 (14.4)	38 (18.8)	34 (16.8)

### Sexual distress

The distress scores of the spouses of patients with cervical cancer with respect to sexual health ranged from 8 to 35 (27.24 ± 7.88), and the degree of sexual distress of the spouses in the study sample is shown in Table 3. The top three questions are as follows: a) You had less or no sexual desire after your spouse was ill (98.5%); b) You and your spouse had difficulty having intercourse or had no sex after your spouse was ill (95.5%); c) After your spouse was sick, you looked up questions about sexual health online, but you were not sure if the information was accurate (95.5%). In addition, most people reported being reluctant to talk about sexual health issues with others (83.7%) and being afraid that sexual distress would affect family relationships (64.4%).

**Table 3:** Sexual distress among the spouses of patients with cervical cancer.

Distress	Agree (%)
You feel that sexual problems are private and awkward to discuss with health care professionals or others	83.7
After your spouse was sick, you looked up questions about sexual health online, but you were not sure if the information was accurate	95.5
You were afraid to discuss sex with your spouse after she was ill	85.1
You had less or no sexual desire after your spouse was ill	98.5
You and your spouse had difficulty having intercourse or had no sex after your spouse was ill	95.5
You were distressed about how to address your sexual needs after your spouse was ill	51.0
A lack of fulfilment in your sexual life after your spouse was ill affected your mood at work and in life	48.5
If you did not have sex with your spouse for a long time, you may worry about the impact on your marital relationship	64.4

### Sexual health needs

The sexual health needs scores of the spouses of patients with cervical cancer ranged from 3 to 12 (8.68 ± 3.64). The scores for one item (“Before or during treatment, your health care provider should tell you that the impact of treatment on your sexual health” ranged from 1 to 8 (7.02 ± 2.12) points. The scores for another item (“At discharge, health care providers should give patients and their families sexual health information and guidance”) ranged from 2 to 6 (5.11 ± 3.03) points. Furthermore, scores for another item (“During follow-up, health care workers should give patients and their families related knowledge and guidance”) ranged from 3 ~ 7 (7.13 ± 5.25) points. The respondents were also asked how they would like medical staff to guide them, and their responses were as follows: a network information consulting platform (50%), private outpatient consultations (30%), and knowledge lectures and health instruction manuals (10%).

### The relationships among sexual cognition, distress and needs

**Fig 1:**
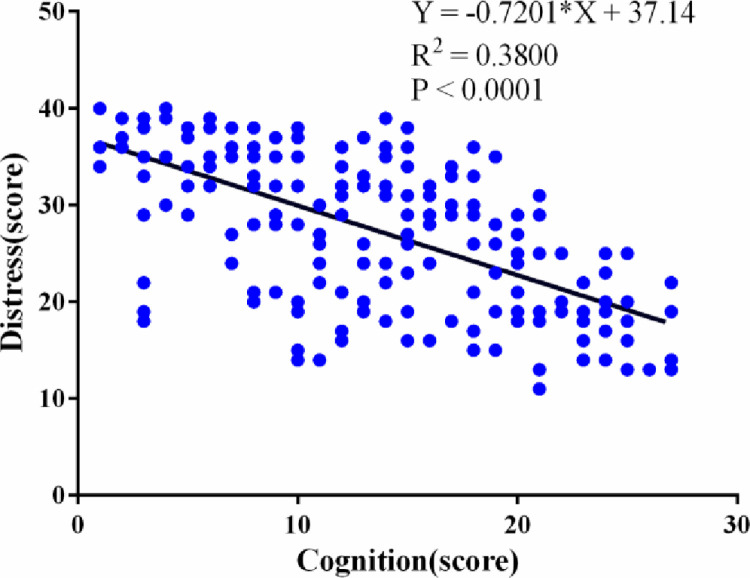
Correlation analysis between sexual cognition scores and distress scores.

**Fig 2:**
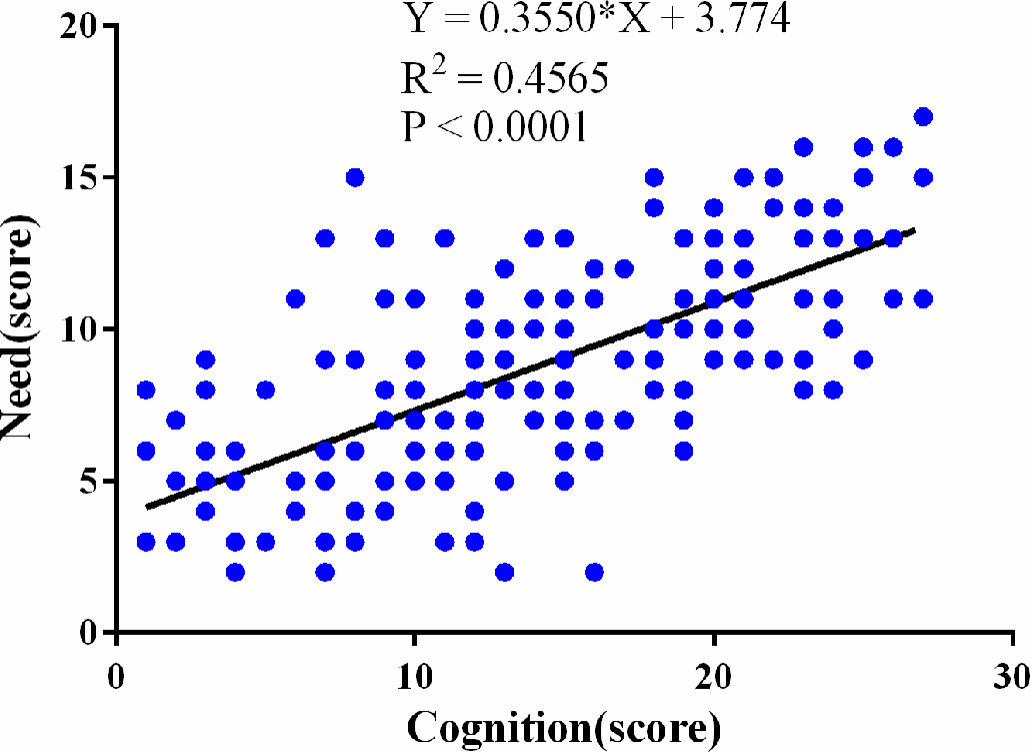
Correlation analysis between sexual cognition scores and needs scores.

**Fig 3:**
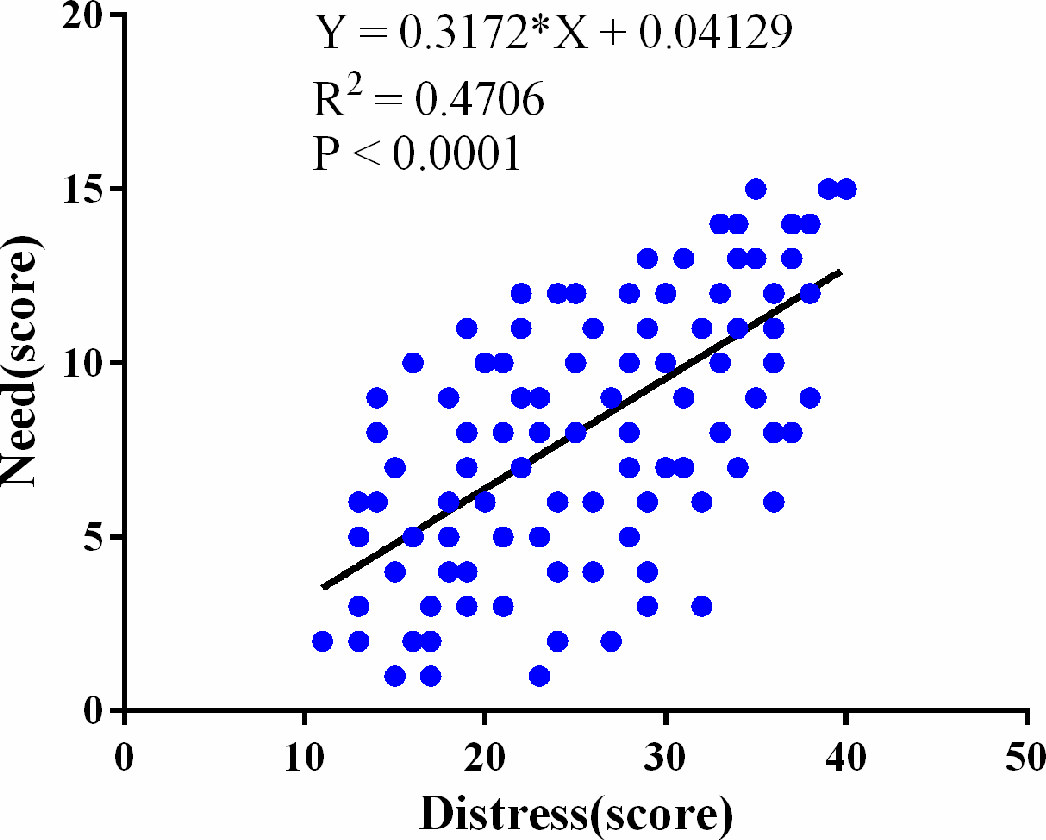
Correlation analysis between distress scores and needs scores.

The results of Pearson’s correlation analysis revealed that there was a negative correlation between sexual cognition and distress (r = - 0.6165, 95% CI: -0.69 to -0.52, P < 0.001) (Fig 1), that cognition was positively correlated with needs (r = 0.6757,95% CI 95% CI: 0.59 to 0.74, P < 0.001) (Fig 2), and that distress was positively correlated with needs (r = 0.6860, 95% CI: 0.60 to 0.75, P < 0.001) (Fig 3). The correlation coefficient matrix of sexual cognition, distress and needs is shown in Table 4.

**Table 4:** Correlation coefficient matrix of sexual cognition, distress and needs.

	Cognition	Distress	Needs
Cognition	1	-0.7201	0.3550
Distress	-0.7201	1	0.3172
Needs	0.3550	0.3172	1

### Factors associated with sexual health cognition

The results from the multiple linear regression analysis are presented in Table 5. Age, education level, place of residence and other factors affect sexual health cognition. Age (β, -0.178; 95% CI, 0.099–1.060), education level (β, 0.152; 95% CI, 0.021–0.663), place of residence (β, 0.665; 95% CI, 0.102–5.789), occupation type (β, 0.507; 95% CI, 0.485–4.982), and monthly family income (β, 0.229; 95% CI, 0.311–1.344) were associated with higher levels of sexual health cognition. The greater an individual’s education level and monthly family income are, the greater his or her understanding of sexual health is. The respondents living in urban areas had greater levels of cognition than did those living in rural areas.

**Table 5:** Multiple linear regression analysis of sexual health cognition.

Variable	B	β	t	P	95% CI
Age, yr (taking ＜30 as a reference)	-0.542	-0.178	-2.177	0.031	(0.099, 1.060)
Education level (taking primary school or below as a reference)	0.331	0.152	2.289	0.023	(0.021, 0.663)
Place of residence (taking urban as a reference)	3.060	0.665	3.767	<0.001	(0.102, 5.789)
Occupation type (taking the office worker as a reference)	2.266	0.507	3.037	0.003	(0.485, 4.982)
Monthly family income, RMB (taking ＜3000 as a reference)	0.788	0.229	2.698	0.008	(0.311, 1.344)

## Discussion

In this study, we found that age, education level, place of residence, occupation type, and monthly family income were associated with higher levels of sexual health cognition. Previous studies [30,31] support the idea that age, education level, and occupation affect sexual cognition. The higher an individual’s education level and monthly family income are, the greater his or her understanding of sexual health. Furthermore, individuals living in urban areas had higher levels of cognition than those living in rural areas did. These findings suggest that we should pay attention to the spouses of patients with cervical cancer, especially spouses with low levels of education, those with low incomes and those who live in rural areas.

Other studies [32,33] have examined the impact of place of residence and monthly income on cognition. In our study, we found that those living in urban areas had higher levels of cognition than those living in rural areas did. The area of residence might reflect the availability of healthcare resources. Rural areas facing medical workforce shortages force residents to choose from a limited number of healthcare providers and services in these areas [34]. Especially in China, owing to the influence of traditional culture and the shortage of medical resources in rural areas, most patients and their spouses believe that the most important thing is the treatment of the disease, and sexual health is not the main reason for them to seek medical treatment. In addition, they think it is awkward to talk to medical staff about sex, and they have insufficient financial resources to pay for sexual health treatment [19].

Thus, we suggest that nurses should conduct a comprehensive assessment of spouses, including evaluations of the place of residence and the financial situation of the family, to provide targeted sexual health guidance to improve their spouses’ sexual cognition. Additionally, the national health department should improve the provision of rural medical care to meet the needs of rural residents.

The findings of this study revealed that there was a high degree of sexual distress among the spouses of patients with cervical cancer. The influence of traditional Chinese culture and patients’ lack of postoperative sexual health knowledge may explain this phenomenon. Chinese people are usually viewed as sexually conservative, and there is still a lack of knowledge about sexuality [35,36]. Most patients and their spouses believe that having sex after surgery can be harmful and even lead to a recurrence of the disease, and the spouse is physically and mentally exhausted from caring for the patient for a long time, so they have less or no sexual desire after the wife becomes ill [21].

In addition, after cancer treatment, common changes in the female body include shortening and narrowing of the vagina and reduced vaginal lubrication, which can strongly affect intercourse [37]. Some couples resume their sexual life after cancer treatment, but owing to changes in body structure, they do not experience the same degree of pleasure as before, and many patients and their spouses believe that if they resume their sexual life, it will lead to mutual transmission [21,38].

Most people are reluctant to talk about sexual health issues with others, which is consistent with previous research showing that Chinese people’s references to sexual issues are often vague and that only a few people express their views about bodily attraction or describe concrete sexual behaviour [19,39].

Cancer and its treatment pose challenges that affect not only patients but also their significant others, including intimate partners. Accumulating evidence suggests that couples’ ability to communicate effectively plays a major role in the psychological adjustment of both individuals and the quality of their relationships [40]. In this study, we found that spouses’ fear that sexual distress will affect their family relationships, and one previous study [41] supported this idea. In addition, another previous study reported that sexual issues may lead to emotional distancing between patients and their spouses and harm their relationships, and women with cervical cancer had a 40% higher divorce rate than women with other cancers did [42].

Therefore, health care professionals should increase their awareness of sexual issues, and sex-related issues should be routinely addressed. We should actively provide psychological counselling regarding sexual health and guidance to the spouses of patients with cervical cancer. In addition, changing a spouse’s negative view of sex can enable them to resume sexual activity earlier, thereby improving the couple’s relationship and quality of life.

The findings of our study revealed that there was a negative correlation between sexual cognition and sexual distress. This correlation is likely due to spouses and patients knowing how to solve their problems after gaining knowledge about sexual health. However, few similar studies exist, most of which have suggested that sexual life for couples with gynaecological cancer should be a part of regular care by all health care providers [42]. However, there is less research on how to improve a partner’s sexual awareness; therefore, more in-depth research on this topic should be conducted in the future to determine whether improvements in sexual health cognition can actually reduce sexual health distress.

This study suggested that the more distress spouses experience, the greater their need for knowledge about sexual health, and network information consulting platforms are common sources of such information for spouses. The lives and relationships of many patients and their partners are negatively affected by sexual dysfunction, and receiving information and practical advice is the most widely available form of psychosexual support for partners [37,43]. This finding is consistent with our findings that patients are motivated to seek help because they are chronically troubled by sexual distress.

Recently, medical staff in China have gradually begun to devote attention to the sexual health of patients. Previous studies [6,44,45]have reported that the sexual quality of life of patients with cervical cancer after treatment is affected by multiple factors. Guidance on treatment, mental status, and posttreatment sexual life should be offered in an individualized manner to improve the sexual quality of life of these patients. However, these studies did not examine the sexual health management of the patient’s spouse. These findings suggest that nurses should provide relevant knowledge about sexual health and pay attention to the psychological needs of spouses according to their cognitive level and degree of sexual distress.

However, providing spouses with good health care services is a challenge for nurses in China. Most nurses have lower educational levels (i.e., less than 12 years). These nurses lack knowledge on how to cope with the damage to sexual health caused by cancer treatments [42]. Hence, nurses usually cannot meet patients’ or spouses’ needs for psychological and sex-related knowledge.

The results of this study highlight the need to train sexual health professional nurses and build sexual health management teams that can provide professional guidance on sexual health issues for cervical cancer patients and their spouses to maintain their sexual health. However, perhaps due to the influence of traditional Chinese culture, husbands and wives often use network information consultation platforms to obtain sexual health knowledge. Therefore, a special information-based sexual health diagnosis and treatment platform should be established in the future to provide convenient consultation, diagnosis and treatment platforms for patients, husbands and wives.

In this study, we investigated only patients with stage I–III cervical cancer, and the spouses of stage IV patients were not included. In the future, the scope and sample size should be expanded to provide a more reliable basis for guiding the clinical treatment of sexual health.

## Conclusions

The sexual health cognition of spouses of patients with cervical cancer is affected by many factors. When sexual distress is severe, sexual cognition is negatively correlated with pain, and sexual pain is positively correlated with the demand for sexual health knowledge. These results suggest that health care workers should include the sexual health of spouses of cervical cancer patients in routine diagnosis and treatment plans and that nurses should provide targeted sexual health guidance according to the specific conditions of spouses, thereby improving their sexual health cognition, reducing distress, and improving the overall quality of life of patients and spouses.
